# Machine learning within the Parkinson’s progression markers initiative: Review of the current state of affairs

**DOI:** 10.3389/fnagi.2023.1076657

**Published:** 2023-02-13

**Authors:** Raphael T. Gerraty, Allison Provost, Lin Li, Erin Wagner, Magali Haas, Lee Lancashire

**Affiliations:** ^1^Cohen Veterans Bioscience, New York, NY, United States; ^2^PharmaLex, Frederick, MD, United States

**Keywords:** machine learning, Parkinson’s Disease, multi-omic analyses, PD progression, data analysis methods

## Abstract

The Parkinson’s Progression Markers Initiative (PPMI) has collected more than a decade’s worth of longitudinal and multi-modal data from patients, healthy controls, and at-risk individuals, including imaging, clinical, cognitive, and ‘omics’ biospecimens. Such a rich dataset presents unprecedented opportunities for biomarker discovery, patient subtyping, and prognostic prediction, but it also poses challenges that may require the development of novel methodological approaches to solve. In this review, we provide an overview of the application of machine learning methods to analyzing data from the PPMI cohort. We find that there is significant variability in the types of data, models, and validation procedures used across studies, and that much of what makes the PPMI data set unique (multi-modal and longitudinal observations) remains underutilized in most machine learning studies. We review each of these dimensions in detail and provide recommendations for future machine learning work using data from the PPMI cohort.

## Introduction

As part of the drive toward precision medicine, there has been an increased focus on the discovery of biological markers and quantitative techniques to serve as diagnostic and prognostic tools for individual patients, and for monitoring the progression or remission of disease. High-dimensional imaging data, as well as genetics, protein, and other ‘omics’ assays capable of querying genotypes, transcriptomes and interactomes at low cost have opened the possibility of carrying out *de novo* discovery across a vast array of biological variables. These approaches have already begun to generate promising candidate biomarkers for a variety of neurological diseases (Nalls et al., 2014; Sanders et al., 2015; Chang et al., 2017; Wray et al., 2018; Nievergelt et al., 2019), shifting the current paradigm away from candidate studies that have proved to be unreliable and irreproducible for reasons ranging from poor study design to a lack of reporting (Landis et al., 2012; Baker, 2016; McShane, 2017; Scherer, 2017; Sun et al., 2019; Ren et al., 2020) toward profiling whole systems, which will provide a broader perspective for disease understanding.

The increased use of multi-modal and high-dimensional data is readily apparent in recent Parkinson’s Disease (PD) research. PD is the second most common neurodegenerative disorder, reaching up to 4% prevalence by age 80 (Pringsheim et al., 2014). While the proximal mechanisms of PD are well understood to be damage to midbrain dopaminergic neurons, and the underlying genetic causes have been discovered in some cases, most PD diagnoses are idiopathic, and little is known about variation in patient trajectories. One prominent example of research aimed at better understanding PD is the landmark Parkinson’s Progression Markers Initiative (PPMI; Marek et al., 2011, 2018), which since its inception in 2010, has collected longitudinal data across several patient cohorts and data modalities, including

- clinical measures
- brain imaging
- gene expression levels, protein abundance, and genomic variant status
- sensors and wearable devices data

with the aim of identifying biomarkers to support the development of new interventions for PD. This initiative represents an extremely rich and well-annotated dataset for studying the progression of multiple biological variables alongside clinical measures of disease severity.

Broader investigation of whole ‘omic’ domains creates its own set of challenges, however. Classical statistical methods for parameter estimation, classification, clustering, and controlling for false positives may perform poorly or be computationally or analytically intractable in these settings, in which observations are often high dimensional and correlated, and meaningful associations are sparse. These problems are compounded by the increasing realization that to understand complex phenotypes, multiple high-dimensional data modalities will need to be integrated.

With increasing volumes of data being collected, machine learning (ML) techniques offer up a potential solution to the above challenges and are beginning to have an impact on research and healthcare. Here, elements of clinical diagnosis and prognosis are being automated with increasing levels of complexity and accuracy. These techniques may lead to the discovery of novel biomarkers, shedding light on the determinants of disease severity for complex neurological diseases such as PD that affect multiple biological systems and whose etiology is not fully understood. While we focus on machine learning in this review, we note that this approach is not always preferrable to classical statistical methods. In cases with a small number of variables and reasonable, well-defined null hypotheses, a significance testing framework may make sense. Furthermore, as we note below, the line between classical statistics and machine learning is often a blurry one. We also note that machine learning algorithms are not a panacea, and that complex problems will generally require good theory and good data to solve. Some measurements may not completely capture the underlying construct they are designed to. To accurately and meaningfully predict psychosis symptoms, for example, a scale which accurately tracks those symptoms is an obvious requirement.

The extensive collection and analysis of longitudinal data has highlighted the heterogenous nature of PD in general, and of disease progression in particular, where people with PD exhibit varying courses of the disease over time (i.e., individuals with PD can be differentiated along numerous axes, symptom patterns etc.; Marquand et al., 2016; Feczko et al., 2019). Akin to the “curse of dimensionality” in machine learning that describes the exponential need for more data as the dimensionality of the feature space grows (Bellman, 1956), this “curse of heterogeneity” presents multiple challenges to the field with respect to the increased sample size required to power discovery efforts that define homogeneous subgroups within a heterogeneous population who share a common clinical diagnosis. Developing tools to disentangle this heterogeneity, and to therefore subtype patients in clinically meaningful ways, will be useful for trial design and clinical care, an area with high rates of failure (Arrowsmith and Miller, 2013; Harrison, 2016) partly due to a lack of insight into the underlying pathology of these disorders of the brain (Krystal and State, 2014).

In this review, we highlight the application of machine learning to the study of PD, focusing our attention on studies using the PPMI data set. In particular, we will consider an approach to be an example of machine learning if it has an overt focus on developing or testing algorithms for *prediction* of diagnosis, symptoms, or progression in unseen data; or for the automated *compression* of high dimensional patient data into lower dimensional factors or clusters. While there are analogs of each of these areas in classical statistical approaches, they represent the main focus of most machine learning research, in contrast to the common statistical goals of null hypothesis testing and parameter estimation. Our aims in this review are to provide a qualitative summary of the research that has been done using machine learning within the PPMI cohort, as well as a reference for researchers interested in continuing the exploration of the cohort to predict diagnosis, symptoms, disease subtypes, risk factors, and patient trajectories in PD.

Machine learning techniques are usually divided into supervised and unsupervised categories. Supervised models are focused on the problem of prediction: they learn a function mapping between some input variables and a target output of interest, such as diagnosis or scores on a symptoms scale. Unsupervised models are focused on compression: they learn to reduce input data so that it has fewer dimensions, based on similarities or redundancies within the input data itself. Unsupervised learning can be further divided into *clustering* or *latent variable* algorithms, depending on whether the data are reduced to categories containing similar sets of observations (often called “subtypes”) or to a set of continuous scores which reflect combinations of the original measurements. Other categories of machine learning, such as semi-supervised learning or reinforcement learning, are currently rare in Parkinson’s research and thus outside the scope of this review.

### PPMI machine learning studies

**Figure 1:**
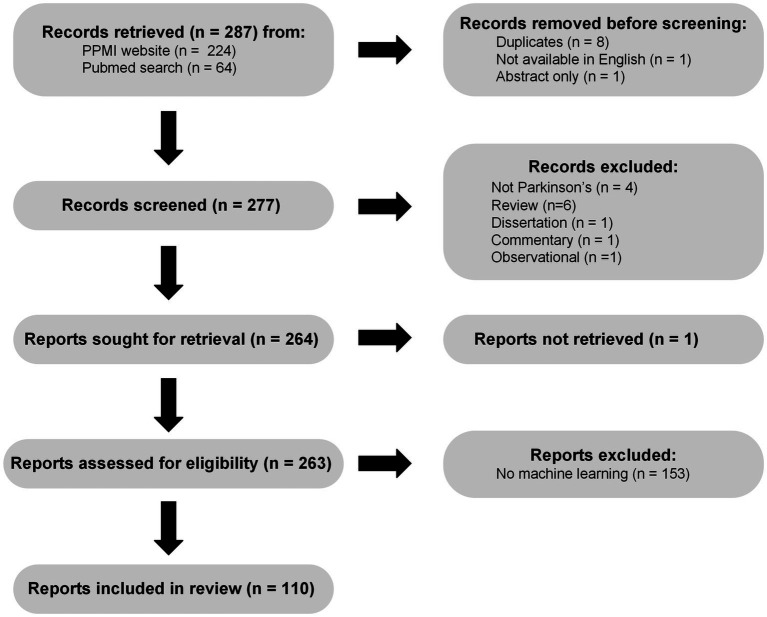
PRISMA (Page et al., 2021) flow chart illustrating the process of searching for, screening, and assessing machine learning PPMI papers.

Based on a review of publications collected on the PPMI website^1^ as of July 5th 2022, combined with a PubMed search for keywords “PPMI,” “Parkinson’s Disease,” “Machine Learning” on the same day a total of 277 unique publications available in English were screened. Figure 1 shows a flow chart describing the selection and filtering of papers, in accordance with PRISMA 2020 guidelines (Page et al., 2021). Briefly, we excluded 1 commentary, 1 observational case study, 1 dissertation, 4 non-Parkinson’s papers, and 6 reviews from further analysis. Of the 263 remaining papers we were able to retrieve, 149 did not meet our criteria for machine learning, because they did not focus on either prediction (supervised learning) or compression (unsupervised learning), leaving a total of 114 machine learning PPMI papers. This large reduction is due in part to the common use of the term prediction in studies where authors perform classical null hypothesis testing, asking essentially whether a particular association or difference is stronger than one would expect due to chance, given a set of assumptions about how the data were generated. This diverges from the intuitive definition of prediction, which involves at a minimum generating (and ideally validating) estimates of some aspect of yet unseen data, which is the criteria we use here. A further 4 studies involved prediction without any model development or fitting, leaving a total of 110 papers which we consider machine learning using PPMI data. The machine learning studies we reviewed varied along several dimensions, three of which we focus on here: (1) the types of supervised or unsupervised models used, (2) the data modalities included as inputs and outputs of the models, and (3) the methods used for model validation. Broadly, most studies used supervised classification or regression methods for characterizing PD and relating different biomarkers and clinical measures to diagnosis, symptoms, and disease progression.

Studies explored a wide range of input data types, including clinical data, imaging, cerebrospinal fluid (CSF) and blood protein levels, as well as DNA and RNA data, to predict disease characteristics or identify latent dimensions or categories, but most used only clinical measures and a small subset of the available neuroimaging data. Studies also varied widely in whether and how they validated their models and performance estimates, an essential step toward making findings reproducible, generalizable, and ultimately relevant to the clinic. We will explore each of these dimensions below, summarizing previous research and making suggestions for improving future work.

### Types of models

Of the 110 machine learning studies we reviewed, almost 90% (97 studies) reported results of supervised models, while only 19 studies used unsupervised methods. Of the papers reporting supervised methods, 55 attempted to predict Parkinson’s diagnosis, representing a majority not only of supervised papers, but of all machine learning papers we reviewed. While early diagnosis of PD, especially in prodromal individuals exhibiting sub-threshold motor symptoms, olfactory deficits, or Rapid Eye Movement (REM) sleep disorder, is an important goal, there are numerous well-validated clinical tools for diagnosing PD at the stage in which patients are enrolled into the PPMI study. Machine learning algorithms focused on diagnosis will have little to say about the major goals of PPMI: to understand variability in patient symptoms, and especially variability in the trajectory of symptoms over time.

Significantly fewer machine learning papers made use of the longitudinal structure of PPMI data, with 26 reports predicting future symptoms from some baseline (which we call “progression prediction”). Because understanding the biological variables associated with heterogeneity in patient trajectories is a core goal of the PPMI project, we summarize these progression prediction papers in Table 1. These studies illustrate many of the characteristics of (and issues with) the broader group of papers we reviewed here, which are discussed in detail below.

**Table 1:** Machine learning papers predicting symptom progression using the Parkinson’s Progression Markers Initiative data set.

Study	Input data					Outcome measures	Modeling approach	Validation method	Summary
	C	D	R	B	I				
1. Adams et al. (2021)	✓				✓	UPDRS III at year 4	CNN	Ten-fold CV	Trained a CNN to predict motor symptoms at year 4 from baseline raw DAT images and year 0 and 1 UPDRS III.
2. Chahine et al. (Chahine et al., 2021)	✓				✓	Diagnosis of α-synucleinopathy (aSN)	Cox hazard regression	None	Tests DAT SBR at baseline for predicting future diagnosis of PD, Lewy Body Dementia, or Multiple System Atrophy. Reports sensitivity and specificity but no validation.
3. Chen et al. (2021)	✓	✓		✓	✓	MCI diagnosis in patients with REM disorder	LASSO, Cox hazard regression	Validation set	Predicted time to MCI from baseline genetic, CSF, DAT, and clinical features.
4. Combs et al. (2021)	✓					Cognition (neuropsychological tests) at year 1	Stepwise regression	Validation set	Prediction of cognitive performance from baseline clinical and cognitive scores in both controls and patients.
5. D’Cruz et al. (2021)	✓				✓	FoG at year 2	Vertex-based shape analysis stepwise logistic regression	None (statistical tests of a subset of features in PPMI data set)	Note: Model was fit to internal cohort. Baseline clinical and MRI features used to predict FoG. Variables selected based on significance and stepwise regression. AUC reported but not validated. Statistical tests of MRI features performed on PPMI data.
6. Faghri et al. (2018)	✓					UPDRS progression sub-type	Nonnegative Matrix Factorization, Gaussian Mixture Models, Random forests	Five-fold CV Test set External validation set	Combined unsupervised and supervised methods to divide patients into progression sub-types and predict sub-type score from baseline clinical measures
7. Gramotnev et al. (2019)	✓	✓		✓	✓	Rate of MoCA change	Logistic regression with variable selection based on relative importance	Monte Carlo (after selecting significant features*)	Used baseline clinical, genetic, DAT, and CSF to predict rate of cognitive decline. Variables selected based on performance before validation*.
8. Gu et al. (2020)	✓			✓	✓	Geriatric Depression Scale at year 2	XGBoost, Stepwise logistic regression	Ten-fold CV for hyperparameter tuning Validation set	Compared methods for predicting depression severity at year 2 from clinical, CSF, and DAT measures.
9. Hayete et al. (2017)	✓	✓			✓	Rate of change in UPDRS II + III and MoCA	Dynamic Bayesian graphical model	None (Statistical tests of a subset of features in PPMI data set)	Note: Model was fit to LAB-PD data. PPMI was used for limited external validation. Predicted motor and cognitive progression mainly at years 5-7 of follow-up. Direct predictive validation was not performed, but a subset of findings was tested statistically in PPMI.
10. Jackson et al. (2021)	✓				✓	Change in UPDRS III at year 1	Ridge regression	External validation set (PPMI)	Predicted motor decline at 1 year from baseline clinical and DAT factors. Note: trained on placebo arm of clinical trial, tested on PPMI
11. Kim et al. (2019)	✓			✓	✓	Freezing of Gait (FoG from UPDRS) at year 4	Cox hazard regression	None	Predicted future freezing of gait, from baseline clinical measures, CSF, and DAT. Reports AUC but no validation.
12. Kim and Jeon (2021)	✓			✓		FoG (UPDRS) up to year 8	NA (ROC analysis of NFL)	None	Predicted gait freezing using serum NFL. Reports AUC but no validation.
13. Latourelle et al. (2017)	✓	✓		✓	✓	Rates of motor and daily living symptoms (combined UPDRS II and III totals, rates estimated from linear mixed-effects models)	Reverse engineering and forward simulation (REFS) model ensemble	Five-fold CV External validation set (LABS-PD)	Large-scale prediction of symptom progression using model ensemble trained on >17,000 SNPs, clinical variables, and DAT and CSF features. <4% variance explained by biological variables. Tested results on external cohort
14. Ma et al. (2021)	✓			✓		UPDRS III at multiple years	Multiple ML models (LASSO, ridge, random forests, gradient boosting) with recursive feature elimination	CV within training set for variable selection validation set	Compared performance of multiple ML models in using each year’s clinical and CSF measures to predict subsequent year’s motor scores
15. Nguyen et al. (2020)	✓			✓	✓	UPDRS III total and MoCA scores at years 1, 2, 3, and 4	Deformation-based morphometry, DNN autoencoder	Five-fold CV (after selecting significant regions*)	Predicted motor and cognitive deficits from baseline MRI, CSF, and clinical scores in patients with REM disorder. Variables selected based on performance before validation*.
16. Rahmim et al. (2017)	✓				✓	UPDRS III at year 4	Random forests	LOO	Year 1 and 2 DAT, MRI, and clinical scores were used to predict UPDRS III total at year 4
17. Ren et al. (2021)	✓					Hoehn & Yahr score	Multivariate functional PCA, Cox hazard regression	External validation set (LABS-PD)	Combined unsupervised dimensionality reduction of clinical and cognitive variables with prediction of functional outcomes.
18. Rutten et al. (2017)	✓				✓	2-year change in anxiety (STAI)	Linear mixed-effects model with stepwise selection procedure	None	Stepwise selection of features for linear mixed-effects model predicting 2-year change in STAI scores from baseline clinical scores and DAT features. No validation of selected features was performed.
19. Salmanpour et al. (2019)	✓					MoCA at year 4	DNNs, LASSO, random forest, ridge, others	Monte Carlo Test set	Tested future cognitive score from a large set of feature selection and prediction algorithms. Genetic algorithm combined with local linear trees performed the best.
20. Schrag et al. (2017)	✓	✓		✓	✓	Cognition (MoCA) at 2 years	Logistic regression with variables pre-filtered based on significance	Monte Carlo Ten-fold CV validation set all after selecting significant features*	Predicted cognitive impairment with clinical scores, CSF, APOE status, and DAT. Selected variables before validation*.
21. Simuni et al. (2016)	✓			✓	✓	Time to initiation of symptomatic treatment	Random survival forest	CV	Predicted time to initiation of symptomatic therapy using random survival forests. No biological variables increased accuracy of prediction above clinical baseline.
22. Tang et al. (2019)	✓				✓	UPDRS III at year 4	DNN	LOO (after selecting significant features*)	Artificial neural network to predict future motor symptoms from imaging and clinical features. Variables selected based on performance before validation*.
23. Tang et al. (2021)	✓			✓	✓	Cognitive decline (MoCA or neuropsychological test scores)	LASSO, Cox hazard Regression	Validation set (*t*-tests to remove variables that differ between training and validation sets*)	Prediction of cognitive decline using baseline clinical, CSF, and MRI features. Features were filtered after training based on similarity between training and test sets*
24. Tsiouris et al. (2017)	✓	✓		✓	✓	Rate of change in UPDRS total up to year 2	RIPPER (Cohen, 1995)	Ten-fold CV	Predicted change in UPDRS scores at 2-and 4-year epochs after selecting from over 600 baseline variables, including genetic, CSF, clinical and imaging features
25. Tsiouris et al. (2020)	✓	✓		✓	✓	Rate of change in UPDRS total up to year 4	Naïve Bayes, RIPPER	Ten-fold CV	Extended (Tsiouris et al., 2017) to 4 year follow up
26. Zeighami et al. (2019)	✓				✓	Change in Global Composite Outcome (Fereshtehnejad et al., 2015)	Voxel-based Morphometry, Independent Component Analysis	Ten-fold CV (after selecting significant voxels)	Tested whether baseline MRI-based atrophy marker could predict change in overall severity. Cross-cohort validation prevented data leakage.

Because of the focus of PPMI on variation in symptom trajectories, we summarize here the 26 machine learning papers from our literature search attempting to predict future symptoms. Input Data: C, clinical/demographic; D, DNA/genotype; R, RNA; B, biomarker/biospecimen; I, imaging; CV, cross-validation; LOO, leave-one-out; UPDRS, Unified Parkinson’s Disease Rating Scale; DNN, deep neural network; CNN, convolutional neural network; MCI, Mild Cognitive Impairment; NFL, Neurofilament light chain; *potential data leakage. In papers containing both null hypothesis tests and measures of predictive accuracy, only variables and methods included in predictive tests are considered here.

Thirteen additional studies focused on predicting symptoms measured at the same time as the predictive features, while five studies focused on predicting either neuroimaging results or medication state rather than symptoms or diagnosis (Simuni et al., 2016; Freeze et al., 2018; Valmarska et al., 2018; Shu et al., 2020; Lim et al., 2021). One study reported predictive accuracy for both diagnosis and symptom levels (Soltaninejad et al., 2019). The most popular supervised models were linear regression (often with a L1-norm regularization penalty on coefficients to promote sparsity, called LASSO (Tibshirani, 1996)), support vector machines (SVM) (Cortes and Vapnik, 1995), and random forests (RFs; Breiman, 2001) and/or gradient boosting methods (Friedman et al., 2000; Friedman, 2001).

A smaller number of studies used unsupervised learning to generate latent variables or clusters to capture patient variability. Of the 19 studies using unsupervised methods, 11 were concerned with sub-typing Parkinson’s patients using clustering models. Eleven used latent variable or dimensionality reduction methods with continuous latent factors, and 3 used both sub-typing and continuous latent variables.

Only 6 papers combined supervised and unsupervised methods. This is surprising given the stated focus of much PD research on finding subtypes which can predict differential progression across groups of Parkinson’s patients. To discover latent sub-groups of patients and find predictors of future sub-group membership, it is likely that supervised and unsupervised models will need to be integrated. Notably, 3 papers combined clustering of patients into subtypes with prediction of current or future symptoms. In one example, Faghri et al. (2018) used an unsupervised combination of Nonnegative Matrix Factorization (NMF) and Gaussian Mixture Models (GMMs), respectively, to reduce dimensionality and cluster patients into sub-types, and RFs for supervised prediction of symptom levels 4 years later. Valmarska et al. (2018) explored the impact of baseline motor symptoms on disease progression. They used an unsupervised clustering approach to group the patients according to the Movement Disorder Society Unified Parkinson’s Disease Rating Scale (MDS-UPDRS) part III scores (Movement Disorder Society Task Force on Rating Scales for Parkinson’s Disease, 2003), and developed a supervised algorithm to determine which features predicted changes in cluster assignment over time. They identified bradykinesia as the most influential attribute in their model. Zhang et al. (2019) first trained Long Short-Term Memory (LSTM) (Hochreiter and Schmidhuber, 1997) networks to encode sequences of input clinical observations in order to predict a set of target measures, which included clinical and biological variables (such as Dopamine Transporter [DAT] scan and CSF measurements). Next, they used Dynamic Time Warping (DTW; Bellman and Kalaba, 1959) to estimate the similarity between sequences of LSTM activations for each pair of patients. Finally, Student t-distributed Stochastic Neighbor Embedding (t-SNE; Van der Maaten and Hinton, 2008) was used to compress patients into a 2-dimensional space which preserved DTW distances, and patients were divided into three subtypes using k-means clustering in the compressed space. The three clusters differed in terms of age, baseline and slope of motor and cognitive symptoms, as well as DAT scan level decline.

Integrating supervised and unsupervised models should be an increasing focus of future work in order to ensure that we define subtypes or other latent variables that are potentially useful. To this end, research should combine the unsupervised discovery of latent factors or subtypes that explain heterogeneity in patient characteristics with supervised learning to predict latent scores from baseline symptoms and/or to predict future symptoms from latent scores.

### Modalities

#### Clinical data

Unsurprisingly, almost all machine learning studies (96/110) made use of clinical or cognitive variables, including patients’ diagnosis as well as motor, cognitive, psychiatric, and functional/daily-living symptoms. These variables were commonly used as model inputs, targets for prediction (for supervised models), or both. The most common scale used was the UPDRS, which measures patients across all these domains. Other clinical variables included measures of dopaminergic therapy (Valmarska et al., 2018; Weintraub et al., 2020; Lim et al., 2021; Severson et al., 2021) and rapid eye movement (REM) sleep behavior disorder (RBD; Prashanth et al., 2014; Tsiouris et al., 2020; Chen et al., 2021). Seventeen studies used only clinical and demographic variables. For example, the three hybrid clustering-supervised-learning papers we described above used only clinical data as inputs to the clustering model.

Leveraging recent advances in deep learning, de Jong et al. (2019) presented a new method (VaDER) that combined an LSTM and a variational autoencoder (Kingma and Welling, 2013) to model multivariate clinical time series data. This method produced clusters with clinically divergent disease progression profiles. The identified clusters differed in gender, Schwab and England score and symptoms of depression. While they did not externally validate or assess the stability of these clusters (see *Validation and Data Leakage*, below), they did test for statistical differences between clusters in brain areas including the caudate. In the supervised domain, Prashanth et al. (2014) developed a SVM model for predicting PD diagnosis from olfactory deficits (measured by the University of Pennsylvania Smell Identification Test, UPSIT) as well as REM sleep disorder symptoms (measured by the REM Sleep Behavior Disorder Screening Questionnaire, RBDSQ), which reached a sensitivity of 88% and a specificity of 78% on an unseen validation set.

#### Neuroimaging markers

The next most used modality was neuroimaging, with 79 studies using some form of brain imaging data. PPMI neuroimaging includes structural and resting-state functional MRI, as well as Diffusion Tensor Imaging (DTI) data, and DAT scans. DAT imaging, a SPECT technique which measures dopamine transporter binding, was the most common neuroimaging modality in the machine learning papers we reviewed, specifically measured in the basal ganglia, most often using Striatal Binding Ratio (SBR).

DAT imaging has been extensively utilized as an input biomarker variable across the studies we evaluated. One study demonstrated that the inclusion of DAT features in addition to demographics and clinical measures improved prediction of UPDRS scores at 4 years post-baseline (Rahmim et al., 2017). It may be that DAT imaging represents a distinct domain, or possibly an endophenotype of PD progression. We observed that across these studies, DAT scan features were mainly limited to SBR, although extraction of novel image features has also been performed (Wenzel et al., 2019; Adams et al., 2021; Zhou and Tagare, 2021).

The information DAT imaging yields regarding dopaminergic tone may make it a key component in models of PD. However, it is worth noting that DAT level measurements are currently used in the consensus diagnosis of PD for PPMI subjects. That is, participants are not classified as having PD unless they exhibit a DAT scan deficit, even if they have motor abnormalities associated with the disorder. Because of this, subjects in the PPMI database labeled PD are guaranteed to have lower DAT levels in the basal ganglia than healthy controls or other groups. This is an interesting example of circularity in machine learning evaluation, which we will discuss in more detail below. We urge caution in the use of DAT levels in machine learning models containing PD diagnosis as a variable, especially in supervised models designed to predict diagnosis.

There were other examples utilizing imaging features from structural, functional, or DTI MRI scans (Badea et al., 2017; Peng et al., 2017; Amoroso et al., 2018; Singh et al., 2018; Won et al., 2019; Chan et al., 2022). One study by Uribe et al. used unsupervised hierarchical cluster analysis of structural MRI data to produce subtypes of untreated PD patients based on cortical atrophy patterns at baseline (Uribe et al., 2018). Here, two subtypes were identified, both of which were characterized by cortical thinning when compared to healthy controls. One of the subgroups showed more pronounced cortical atrophy and exhibited lower cognition scores. In a supervised learning study, Zhang et al. (2018) used a Graph Neural Network to classify patients vs. controls using DTI data.

However, such studies were relatively rare. One potential reason for this is that while other sources of data are provided in tabular form and require little preprocessing, MRI images require extensive processing and feature engineering before they can be incorporated into most machine learning models [convolution neural networks, which can work with raw or preprocessed images directly, are a notable exception and have been used in some PPMI studies (Yagis et al., 2021)]. Opportunities thus exist to further explore the utility of imaging markers. However, caution is warranted, and MRI preprocessing steps should be validated and standardized moving forward. Testing different DTI preprocessing pipelines on PPMI data, for example, Mishra et al. (2019) concluded that results are heavily dependent on the choice of preprocessing and analysis.

#### Genetic and other biomarkers

**Figure 2:**
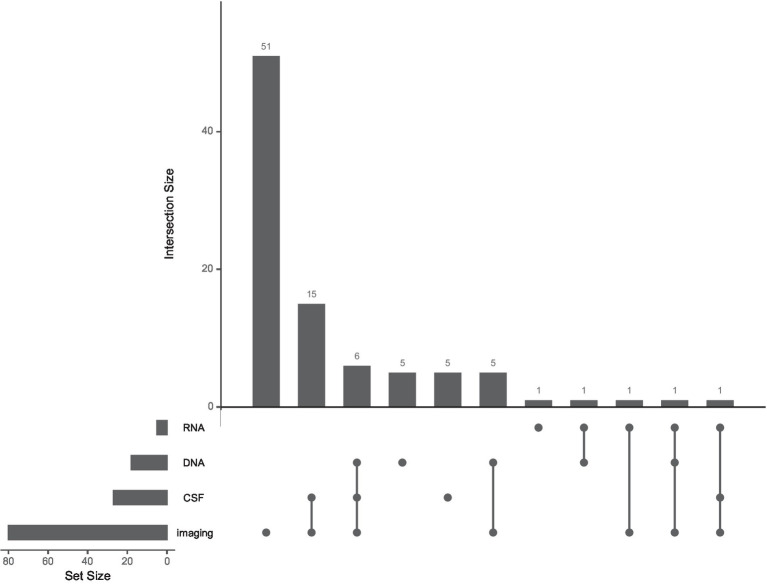
The use of multiple biological modalities is rare in machine learning studies of Parkinson’s Progression Markers Initiative data. UpSet plot (Lex et al., 2014; Conway et al., 2017) showing frequencies of different combinations of imaging, CSF, DNA, and RNA data in the machine learning studies we reviewed. Each set of connected dots represents a combination of modalities (bottom). Intersection size is plotted as the number of studies for a particular modality combination (top). The set size is plotted as the overall number of studies using each modality (left). Ninety-two papers used at least one biological modality, including Dopamine Transport imaging striatal binding ratio scores. Thirty studies (out of 110 total) combined multiple biological modalities.

Relatively few machine learning studies used information from DNA (18 studies), RNA (5 studies), or CSF-based biomarker (27 studies) measurements. Forty-one studies made use of at least one such biomarker, while only 10 used multiple (see Figure 2 and *Multi-modal markers*, below).

Using CSF measures as well as non-motor clinical scores, Leger et al. (2020) compared several different supervised machine learning techniques to classify participants as PD, healthy controls, or subjects without evidence of dopamine deficiency (SWEDD). Gradient boosting decision trees (XGBoost, Chen et al., 2015) performed the best at distinguishing these classes, with an area-under-the-receiver-operating-curve (AUC) of 0.92 for PD patients versus healthy controls and 0.82 for patients versus SWEDDs. CSF alpha-synuclein was an important feature for distinguishing PD patients from controls.

Weintraub et al. (2022) built a model predicting impulse control disorders in Parkinson’s patients, combining DNA from a genome wide association study (GWAS), genes previously implicated in impulse control disorders, and clinical variables to separate patients into low and high impulse control groups, with moderate accuracy (AUC of 0.72). Cope et al. (2021) developed a novel algorithm to generate a knowledge graph based on prior literature and known protein co-expression patterns, which is used to select DNA features and multiplicative interactions between features for use in standard machine learning algorithms. This method outperformed standard polygenetic risk scores in detecting PD (AUC = 0.85), highlighting potential interactions between known genetic risk loci and pathways.

RNA was the most infrequently used modality in the machine learning papers we reviewed. In one rare example using blood-based RNA sequencing, Pantaleo et al. (2022) used XGBoost models to classify subjects as PD patients or healthy controls from baseline gene expression data. The approach reached moderate accuracy (AUC of 0.72) and generated a large number of potential genes and expression pathways to be validated and explored in future work.

The limited number of papers using DNA, RNA, and CSF protein measures highlights the fact that these non-clinical data have been under-utilized to date in PPMI machine learning reports. These data modalities represent a potentially untapped resource and should be a major focus of future work.

#### Multi-modal markers

One of the most promising aspects of PPMI is the potential to combine multiple modalities to gain a broader perspective on biological mechanisms of patient heterogeneity. A majority of the studies we reviewed made some use of multiple modalities, although many of these used a single biological modality to predict clinical diagnosis. Many others combined clinical scores with DAT SBR alone. Relatively few studies have taken advantage of the breadth of biological data made available as part of PPMI. Of the 41 studies which made use of either DNA, RNA, or CSF measures, for example, no study used all three. In general, few studies (30/110) integrated multiple biological modalities (imaging, DNA, RNA, or CSF). Figure 2 shows the frequency of each biological modality as well as of multimodal combinations in the literature we reviewed. As for neuroimaging, most multimodal studies with brain imaging used DAT scan measurements in the basal ganglia, with fewer using structural or functional MRI or DTI.

In one example of multimodal analysis of symptom progression, Tsiouris et al. (2017) developed a supervised algorithm for selecting and combining subsets of features based on their discriminative ability, which they combined with Naïve Bayes (Domingos and Pazzani, 1997) and Repeated Incremental Pruning to Produce Error Reduction (RIPPER; Cohen, 1995) classifiers. They trained their feature selection algorithm and classifier to predict which category of rapid progression (measured as the quantile of the slope of UPDRS I-III total score) patients would fall into at 2-and 4-year epochs, using around 600 baseline features including selected SNPs, CSF-and blood-based biomarkers, clinical scores, and DAT and MRI features.

In another study or motor symptom progression, Latourelle et al. (2017) used a Bayesian machine learning based approach known as Reverse Engineering and Forward Simulation to form an ensemble of supervised models to predict symptom trajectory from genetic (53 pre-specified SNPs, PCs as well as a relatively large number (>17 K) of SNPs after pruning for linkage disequilibrium) and baseline CSF and clinical variables. Results showed baseline motor function, sex, age, DNA, and CSF biomarkers to be predictors distinguishing slow and fast progressors (27% of variance explained in PD patients in leave-one-out cross-validation). Notably, the contributions of biological variables were relatively small, with all SNPs explaining <3% of variance in progression and CSF measures explaining <0.5%. They also tested the model ensemble in an external non-PPMI cohort (LABS-PD; Ravina et al., 2009), where it showed reduced, but significant, performance (9% of progression variance explained). Notably, there was no overlap between the biological features reported as important in this model and the Tsiouris et al. model described above, indicating that there is much more work to be done in studying the stability of machine learning findings in PPMI.

Multimodal models were also used to predict progression outside of the motor domain. In an example focused on cognitive decline, Schrag et al. (2017) built a logistic regression model containing features from DAT imaging, Montreal Cognitive Assessment (MoCA), UPSIT, and CSF Abeta/Tau ratio as predictors of cognitive impairment in PD 2 years from baseline. They reported robust performance determined by AUC of ~0.8, higher when including biological variables compared to using age alone. However, this study seems to have filtered features based on their ability to differentiate cognitively impaired subjects before performing cross-validation or splitting data into training and test sets (see *Validation and data leakage*, below), which calls the accuracy of these performance estimates into question.

Unsupervised learning of multiple modalities can be difficult, in part because high-dimensional modalities can dominate contributions to latent dimensions or classes. Using an approach known as similarity network fusion (SNF), Markello et al. (2021) were able to combine imaging, clinical, and CSF data modalities available in the PPMI cohort. They showed that this approach better captures similarities between patients across modalities with different dimensions than standard approaches of concatenating data across modalities. The fused similarity networks can then be decomposed *via* clustering or continuous latent factor algorithms. Interestingly, they tested both approaches in this paper, comparing diffusion embedding (continuous axes) with spectral clustering (discrete subtypes), two methods for unsupervised learning on graphs. They found that a continuous latent space provided the more parsimonious fit to the data.

One issue which is often overlooked when using biological modalities for prediction is how performance compares to a baseline model with only clinical data. In one cautionary example, Simuni et al. (2016) looked to identify clinical and biological predictors of time to initiation of treatment using RFs. They found that baseline clinical measures were most strongly predictive, and that the inclusion of biomarker data such as P-tau and DAT uptake did not improve predictive performance of the model above this baseline.

For the most part, machine learning papers have so far failed to make use of the uniquely multimodal data available from the PPMI project. Future work should focus on validating algorithms for combining multiple high dimensional modalities and testing whether biological data can improve predictions of patient trajectories above baseline clinical scores. However, the combination of multiple high dimensional sources of data will create problems of its own.

### Validation and data leakage

Because machine learning approaches often use powerful algorithms with more tunable parameters than observations, overfitting is a serious concern, and it is essential that findings be validated by estimating stability and performance on unseen data. This is often done with one or more of the following techniques: Monte Carlo sampling, in which training and test sets are repeatedly randomly sampled; k-fold cross-validation, in which observations are divided into k groups, which are then cycled through with each group serving as the test set for one round of training; or maintaining a separate validation set to test performance only after all modeling decisions have been made. In the case of PPMI, sometimes other PD studies with overlapping observations are used as an external validation set, providing an even more meaningful test of model generalizability. In any case, it is essential that parameters-including hyperparameters such as which features or observations to include, what optimization algorithms to use, or even which particular model out of multiple possibilities is selected-be chosen only after isolating a test set from the fitting process.

Surprisingly, 13 supervised studies of the 97 we reviewed described a measure of predictive accuracy but showed no evidence of having a validation procedure for evaluating it. There are more subtle issues in validating predictive models than simply ignoring validation, however. In many cases, validation is performed in such a manner that information has been “leaked” from test set observations to the model fitting procedure, thus invalidating the validation process (Yagis et al., 2021). In one common form of data leakage, features are selected based on statistical tests (e.g., differentiating PD from healthy controls), before splitting the data into training or test sets to train a classifier to differentiate PD from healthy controls. This is circular, because the variables have already been selected because they distinguish PD from healthy controls across both training and test subjects. Twenty more of the studies we reviewed (in addition to those with no validation method at all) showed evidence of such data leakage.

Overall, the lack of attention paid to model validation and clear separation of training and test sets is concerning. In addition to the 33% of supervised studies described above, in others it was unclear from methods precisely how models were evaluated: at what point in the analysis pipeline training and test sets were separated, or even whether this occurred at all. Without clear descriptions of this process, papers are not reproducible and evaluating their results is challenging.

In unsupervised settings, even less attention is paid to model validation. How exactly to validate unsupervised models can be a difficult question, since there are no labels or target variables to predict. The answer will depend on the exact model being used, and detailed treatment is outside the scope of this review. However, latent factor models with continuous axes can in general be treated like regression models in terms of evaluation and validated the same way (e.g., variance explained or mean squared error on held out data; Owen and Perry, 2009). In principle, clustering methods can be treated similarly and evaluated on held-out data, since many clustering models can be considered special cases of continuous dimensionality reduction models (Fu and Perry, 2020). This seems to be rarely done in practice, and of the 19 studies using unsupervised machine learning we reviewed here, 13 failed to provide any prediction-based validation of their unsupervised learning models. Other studies varied in how they tested stability or validity of their models, with only 4 studies explicitly using held-out data to evaluate.

As noted above, external validation can provide a more stringent test of model generalizability than held-out data. After all, we would like our findings to apply to broader populations of patients than those meeting the criteria for enrollment in a specific study. Datasets such as PARS (Parkinson’s Associated Risk Study), Penn-Udall (Morris K Udall Parkinson’s Disease Research Center of Excellence cohort), and 23andMe were used for external validation by a small number of studies reviewed here. These datasets should be extensively evaluated to understand the similarities and differences with PPMI and their suitability as independent replication/validation datasets, while considering known confounders such as age, sex, and other demographics (Van Den Eeden et al., 2003; Pringsheim et al., 2014; Simuni et al., 2016; Hayete et al., 2017; Latourelle et al., 2017; Rutten et al., 2017; Schrag et al., 2017); which measurements were collected; and differences in preprocessing of biospecimen and imaging data.

Future papers need to present a clear strategy for estimating validity and generalizability of findings. Authors should report how evaluation procedures were kept separate from model training in a clear and reproducible manner. In addition, papers should justify the evaluation metrics chosen for a particular study (sensitivity, specificity, AUC, recall, etc.) based on the costs and benefits of different types of model errors and on any potential issues with specific metrics in a particular setting (e.g., severely imbalanced classes).

## Discussion

One striking aspect of the machine learning PPMI literature we reviewed here is the lack of overlap between findings across studies. This is perhaps unsurprising given how much studies differed in their input features, target variables, and modeling approaches. Based on our review of this variability, we make the following recommendations to improve future PPMI machine learning research:

- One of the major goals of PPMI is to understand why different PD patients have different disease trajectories. Machine learning studies should place more focus on predicting variability in patient symptoms rather than distinguishing PD patients from healthy controls, especially variability in future symptom progression from baseline measurements.
- There is increasing interest in sub-typing or phenotyping PD patients, but we need to ensure that our latent categories or dimensions are consistent, predictable, and useful. Much more work is needed in testing the stability across different unsupervised methods (see Alexander et al., 2021 for an example in Alzheimer’s research), and for validating proposed sub-types. Algorithms should also be developed and validated for combining sub-typing or latent factor discovery with supervised learning of patient outcomes.
- Future work should incorporate the multiple biological modalities available to PPMI researchers. This is especially true of raw MRI and DTI images, DNA, and RNA transcriptomic data, as well as multimodal combinations of these domains. Preprocessing pipelines for each domain should be validated and standardized across studies.
- For longitudinal studies with biological modalities, studies should report the extent to which a model improves upon results using only baseline clinical and demographic information.
- More care needs to be taken to validate machine learning results in PPMI studies. Supervised and unsupervised methods need to be tested on unseen data. Measures of predictive accuracy such as AUC should not be reported on training data alone, especially after filtering variables for significance or prediction improvement. When performing validation, data leakage— often caused by preprocessing or variable selection involving both training and test data sets— needs to be more scrupulously avoided in future work. Finally, these procedures need to be articulated more clearly and in a manner that can be reproduced by other researchers. To suggest truly generalizable results, findings should eventually be validated in data sets external to PPMI.

## Author contributions

LLa conceptualized the work and contributed to the review of articles and writing of manuscript. RG contributed to the review of articles and writing of manuscript. AP, EW, and LLi contributed to the review of articles. MH conceptualized the work. All authors contributed to the article and approved the submitted version.

## Funding

This work was jointly funded by Cohen Veterans Bioscience (COH-0003) and a generous grant from the Michael J. Fox Foundation as part of the Parkinson’s Progression Markers Initiative (PPMI) (MJFF-019075). Data used in the preparation of this article were obtained from the Parkinson’s Progression Markers Initiative (PPMI) database (www.ppmiinfo.org/data). For up-to-date information on the study, visit www.ppmi-info.org. PPMI is a public-private partnership funded by the Michael J. Fox Foundation for Parkinson’s Research and funding partners. The list with full names of all PPMI funding partners found at www.ppmi-info.org/fundingpartners.

## Conflict of interest

The authors declare that the research was conducted in the absence of any commercial or financial relationships that could be construed as a potential conflict of interest.

## Publisher’s note

All claims expressed in this article are solely those of the authors and do not necessarily represent those of their affiliated organizations, or those of the publisher, the editors and the reviewers. Any product that may be evaluated in this article, or claim that may be made by its manufacturer, is not guaranteed or endorsed by the publisher.

## Footnotes

^1^ https://www.ppmi-info.org/publications-presentations/publications/
